# Case of Poisoning by Cantharides

**Published:** 1855-04

**Authors:** 


*Case of Poisoning by Cantharides.*

(Read before the Boston Society for Medical Improvement, by C. D. Homans, M.D.)

The following account was communicated to me in a letter from Dr. C. H. Hildreth, of Gloucester, Mass.

On the 27th of October, at 2, A. M., was called to a patient giving the annexed history and presenting symptoms enumerated below:—

Early in the preceding evening he applied at an apothecary's and purchased about ℥ss. of a powder supposed to be the *pulvis aloes cum canella* of the pharmacopœia, known among the vulgar as *picra*, or, as usually pronounced, *pikery*. The medicine was delivered by a boy in attendance. The patient put the powder into a bottle, added to it a tablespoonful of gin, and shaking the mixture took two spoonfuls, his usual dose for the relief of the irritation of ascarides, from which he was then suffering. He slept as well as usual until 12 o'clock, when he awoke with a severe pain in the lower part of the abdomen, thence extending into the lumbar region, but most intense just above the pubis. This rapidly increased to an alarming degree, and in the course of two hours, at the expiration of which time I saw him, became almost unendurable, although the patient was a man of much fortitude. There was some nausea, but no pain in the stomach, or indeed anywhere except as above mentioned.

Upon examination of the mixture which he had taken, the supposed *picra* proved to be powdered cantharides. Free emesis was immediately produced by the exhibition of the sulphate of zinc and copious dilution with warm water. He vomited several times, the powdered flies being expelled at every repetition of vomiting, but the pain in the abdomen was not in the least relieved. I therefore directed large injections of warm water, frequently repeated, and administered ten grains of camphor and one grain of sulphate of morphine, which dose I repeated every half hour until four doses had been taken, by which time great relief was experienced, and I left the patient.

Three hours after, I saw him again. He had passed water freely; urine natural, and without any trace of blood; had suffered from priapism to an inconvenient extent for a short time, but it had now entirely subsided. Patient was sitting up; the pain was very slight, nor did it again recur. Had suffered no inconvenience from the large doses of morphine.

Four days after, I saw him again. He then complained of pain in all his joints, especially in the knees; his eyes were inflamed and painful. Upon examination, slight effusion was apparent in the knee joints, and some inflammation of the sclerotic, which yielded to simple remedies, or more probably subsided spontaneously. Perspiration emitted a strong cantharidal odor, especially in the axillæ. Ten days after, he was able to resume his work.

There are some points of interest in this case, among which may be noticed—

1st. The length of time, viz., about four hours, which elapsed before any perceptible effect was produced by the cantharides. Is not this analogous to the results of its external application?

2. The apparent want of action upon the stomach, so far as can be inferred from the absence of symptoms.

3. The large quantity of morphine taken without producing narcotism. This, however, is sufficiently often observed in painful diseases of all kinds.

The exact quantity of cantharides actually taken into the stomach, it is of course impossible to estimate. The superstratum of liquid in the bottle containing the mixture, is about one third of the whole contents The same proportion would undoubtedly apply to any portion of the mixture after having been shaken up. The quality of the drug is equally uncertain; it was the remainder of a stock that had been on hand for a considerable period, but still retained vesicatory power. The patient had eaten a very light supper before taking the cantharides—a cup of tea and a peace of bread only.—*Boston Med. and Surg. Journal.*

